# Haptic Compensation in Blind People’s Conceptual Representations

**DOI:** 10.1162/OPMI.a.250

**Published:** 2025-10-17

**Authors:** Laura J. Speed, Eva D. Poort, Tanita P. Duiker, Heidi Baseler, Asifa Majid

**Affiliations:** Centre for Language Studies, Radboud University, Netherlands; Donders Institute for Brain, Cognition, and Behaviour, Radboud University, Nijmegen, Netherlands; Department of Psychology, University of York, UK; Department of Experimental Psychology, University of Oxford, Oxford, UK

**Keywords:** concepts, sensory norms, blindness, sensory experience

## Abstract

Vision is typically dominant in our perception of the world. Such asymmetry is also observed in conceptual representations. This could be driven by perceptual experience or learned from other input, such as language. In this study we tested the role of direct perceptual experience in conceptual representation by investigating the sensory underpinnings of word meanings in blind and sighted individuals. Seventeen early-blind and 17 matched sighted Dutch native speakers rated 100 Dutch nouns for their sensory associations across six modalities (vision, audition, haptic, interoception, gustation, and olfaction) on a 0 (not at all) to 5 (very much) scale. To cover a range of concepts we used five semantic categories thought to be strongly associated with different sensory modalities: animals (vision), instruments (audition), tactile objects (haptics), food (gustation), and odor objects (olfaction). We found no difference between blind and sighted individuals in their ratings of visual associations, suggesting that conceptual associations with vision can be learned indirectly via means beyond direct visual perception. However, blind participants did associate concepts more strongly with haptics than sighted participants for all semantic categories except animals. This is evidence for crossmodal compensation in conceptual representation, in line with enhanced tactile acuity reported elsewhere for blind individuals. Overall, the results point to a role for perceptual experience in conceptual representation, but suggest there are other strategies that can be recruited to learn about perception, supporting hybrid models of semantic representation.

## INTRODUCTION

We learn about the world through our senses, but not all senses are equal. Vision dominates our mental life: visual memory is remarkable (e.g., Cohen et al., 2009; Lawless, 1978; Standing, 1973), visual information dominates when the senses are in competition (Colavita, 1974), and it is even claimed around half of the brain is devoted to processing visual inputs (Palmer, 1999; see also Reilly et al., 2020). At the same time, information from smell and taste is poorly represented: imagery for these senses is weak (Andrade et al., 2014; Arshamian et al., 2020), and they are particularly susceptible to contextual modulation (e.g., Herz & von Clef, 2001; Morrot et al., 2001). Given this, it is not surprising that conceptual representation also draws heavily on visual representations, while smell and taste appear to be less relevant (Speed & Majid, 2020).

When speakers of English, for example, are presented with everyday concepts—such as *apple, shiny*, or *run*—and asked to indicate the extent to which each of these is experienced through the five senses, vision overwhelmingly receives higher ratings than the other senses (e.g., Lynott et al., 2020). Even concepts that would seem to draw heavily on smell and taste—e.g., food concepts—are rated highly on vision. This suggests visual information contributes critically to the representation of many concepts, even those that we might intuitively link strongly to another modality. This dominance could be the result of first-hand sensory experience, as exemplified by the asymmetries in cognition noted above. But direct perception is not our only source of information about the senses. We also learn about the world from language—through conversation, reading, listening to the radio, etc. Critically, language appears to reinforce the self-same visual dominance (San Roque et al., 2015; Winter et al., 2018). This makes it difficult to assess the unique role of perceptual experience in conceptual representation in the ordinary course of events.

There is, however, a potential opportunity to test the contribution of direct perceptual experience—that is, by examining conceptual representation in blind people. Congenitally blind individuals do not have the same visual input as sighted people; but their language input may be similar to that of sighted individuals. Recent evidence shows that congenitally blind children receive largely similar language input compared to matched sighted children, even for words that are highly and exclusively visual (Campbell et al., 2025). If visual dominance in conceptual representation is the product of direct perceptual experience, then blind people should not have visual associations with concepts, and may even show evidence of compensation by having stronger associations with other senses. For example, visual and tactile experience are strongly correlated, and this correlation is seen in sensory ratings of words (Lynott et al., 2020; Speed & Brysbaert, 2022; Speed & Majid, 2017). Tactile information may therefore help scaffold blind people’s concepts for which visual information is lacking. This has been observed for early deaf individuals, who rated their sensory associations with words as higher in all modalities except audition compared to a group of hearing participants (Amenta et al., 2024). There is some evidence that blind people have enhanced recognition and categorization in hearing (Bull et al., 1983), touch (Wong et al., 2011), and smell (Cuevas et al., 2009; although see Majid et al., 2017). Perhaps this perceptual compensation is directly reflected in conceptual representation. Consequently, a blind individual may state that *apple* is experienced more through touch, taste, and smell, with little or no contribution of vision. Yet, blind people might receive sufficient input about vision from language. They may know that *apple* is primarily experienced by sighted people through vision (as indicated by linguistic collocations; e.g., *Annie saw the shiny apple*, Anceresi et al., 2023; Lewis et al., 2019; Ostarek et al., 2019), and rate it as primarily experienced visually. If so, perceptual ratings for object concepts would not vary between blind and sighted individuals.

Studies addressing knowledge of specific visual features of objects and events suggest that blind individuals acquire significant visual knowledge. Blind children start using color words around the same age as sighted children and are aware that objects but not events can have colors (Bedny & Saxe, 2012; Landau, 1983), and (color-)blind individuals show striking similarities to sighted people for color words, for example, they know that orange is more similar to yellow than green (Kim et al., 2019a; Marmor, 1978; Saysani et al., 2018; Shepard & Cooper, 1992). Moreover, Bedny et al. (2019) report no differences between blind and sighted participants in semantic similarity judgements of visual verbs (such as *sparkle* and *shine*). A recent study has also shown that blind people’s judgements of words’ concreteness and imageability is predicted by Image-based Frequency—a measure of the availability of a word’s referent in the visual environment calculated from Flickr images—but to a lesser extent than sighted participants (Petilli & Marelli, 2024). Again, this supports the notion that direct visual experience may not be necessary for conceptual knowledge about vision.

On the other hand, Kim et al. (2019a) found that, although there were global similarities, there were notable disparities between blind and sighted people in how they categorized animals according to their color, size, height, shape, and texture. For example, both groups agreed that elephants and rhinos are larger than bears and cows; but only sighted participants judged bears to be larger than cows. Similarly, while most blind participants in Connolly et al.’s (2007) study achieved high accuracy scores when naming the color of fruits and vegetables, a small subset did not, nor did blind participants use color information when asked to sort fruits and vegetables into groups. This suggests that blind individuals’ knowledge of vision remains limited compared to sighted individuals.

In this study, we seek to explore the perceptual basis of conceptual representation for different types of object categories. Instead of asking about knowledge of specific visual properties of objects (e.g., the color, size, or shape of an object), we aim to explore the extent to which visual information underlies the meaning of concepts in blind individuals, as well as whether or not information from other sensory modalities is used in conceptual representation as a form of sensory compensation. We asked a group of early-blind participants and a group of matched sighted controls to rate a set of words on their sensory associations across six modalities (vision, audition, haptic, interoception, gustation, and olfaction), following the procedure of Lynott et al. (2020). These sensory ratings can be used as a measure of conceptual representation since they can predict behavior on semantic judgments such as property verification (e.g., is *jingling* a property of keys?) (Lynott & Connell, 2009), lexical decision (Connell & Lynott, 2012, 2014; Lynott et al., 2020; Speed & Majid, 2017), and free word association (Dymarska & Connell, 2025). To cover a range of concepts, participants rated words from five semantic categories thought to be strongly associated with different sensory modalities: animals (vision), instruments (audition), tactile objects (haptic), food (gustation), odor objects (olfaction). We had the following hypotheses: (H1) if direct perceptual experience matters, blind participants will rate all concepts lower on vision because they have impoverished visual experience; and (H2) if there is perceptual compensation, then blind people will rate items higher on the other senses, particularly sensory modalities that may be enhanced in blindness (audition, haptic, and olfaction).

## METHOD

This study was pre-registered on the Open Science Framework (https://osf.io/k9j2a).

### Participants

Participants were recruited from a participant pool of blind and sighted participants at the local institute. Seventeen blind participants (*M*_*age*_ = 57.4 years, 12 females, 5 males) and 17 control sighted participants matched to the blind participants on age, gender and education level (*M*_*age*_ = 56.8 years, 12 females, 5 males) took part in the study. The average onset of blindness was 8.4 months of age (*SD* = 11.92). Six blind participants could distinguish light and dark, but had no current visual perception of colors or shapes. All participants were native speakers of Dutch. Table 1 depicts demographics of the blind participants.

**Table 1.** Blind participants demographic information.

Age	Gender	Age of blindness	Residual light perception
43	Female	birth	no
69	Male	birth	yes
51	Female	1 year	no
68	Male	1 year	no
48	Female	birth	no
76	Female	5 years	no
71	Male	birth	yes
65	Female	birth	no
51	Female	birth	no
64	Male	birth	no
61	Female	birth	yes
46	Female	birth	no
42	Female	birth	no
51	Female	birth	yes
43	Female	birth	yes
76	Male	2.5 years	no
51	Female	2.5 years	yes

### Stimuli

To have a broad range of concepts in the study, we selected 20 words per sensory modality (vision, audition, haptic, gustation, olfaction) from the Dutch Sensorimotor Norms (Speed & Brysbaert, 2022) based on their dominant sensory modality (the modality with the highest mean rating). Items were selected from the following semantic categories corresponding to each sensory modality: animals (vision), musical instruments (audition), fabrics or tactile objects (haptic), food (gustation), and odor objects (olfaction) (see Table 2 for full list and Table 3 for average perceptual strength per category). We chose 100 items so that the task was of a reasonable duration for participants. Although words were dominant in one sensory modality, they were all multisensory to some extent, as indicated by modality exclusivity score (*M* = 0.41, *SD* = .10, range = 0.24–0.77), where a score of 0 refers to a completely multimodal concept and a score of 1 refers to a completely unimodal concepts. This means that should conceptual information about vision be absent in blind participants, information in other modalities is available to compensate.

**Table 2.** Selected words by semantic category (English translation in brackets).

Animals (Vision)	Instruments (Audition)	Tactile objects (Haptic)	Food (Gustation)	Odor objects (Olfaction)
*alligator*	*panfluit*	*badmat*	*aardappel*	*aftershave*
(alligator)	(pan flute)	(bathmat)	(potato)	(aftershave)

*bever*	*bel*	*bagage*	*avocado*	*afval*
(beaver)	(bell)	(luggage)	(avocado)	(garbage)

*ooievaar*	*cello*	*beddengoed*	*banaan*	*benzine*
(stork)	(cello)	(bedding)	(banana)	(petrol)

*eekhoorn*	*contrabas*	*boksbal*	*broccoli*	*bleekmiddel*
(squirrel)	(double bass)	(punching bag)	(broccoli)	(bleach)

*schildpad*	*doedelzak*	*donsdeken*	*citroen*	*chloor*
(turtle)	(bagpipes)	(duvet)	(lemon)	(chlorine)

*goudvis*	*sax*	*schuurpapier*	*druif*	*deodorant*
(goldfish)	(sax)	(sandpaper)	(grapes)	(deodorant)

*fazant*	*dwarsfluit*	*fluweel*	*framboos*	*wasmiddel*
(pheasant)	(flute)	(velvet)	(raspberry)	(laundry detergent)

*geit*	*harmonica*	*handtas*	*kiwi*	*gas*
(goat)	(harmonica)	(handbag)	(kiwi)	(gas)

*hert*	*harp*	*keukenpapier*	*kool*	*jasmijn*
(deer)	(harp)	(kitchen paper)	(cabbage)	(jasmine)

*kever*	*klarinet*	*knuffelbeer*	*mandarijn*	*kamille*
(beetle)	(clarinet)	(teddy bear)	(mandarin)	(chamomile)

*kikker*	*klokkenspel*	*lasso*	*mango*	*kerosine*
(frog)	(chimes)	(lasso)	(mango)	(kerosine)

*konijn*	*orgel*	*linnengoed*	*meloen*	*lavendel*
(rabbit)	(organ)	(linen)	(melon)	(lavender)

*pauw*	*piano*	*nylon*	*perzik*	*mest*
(peacock)	(piano)	(nylon)	(peach)	(manure)

*vlinder*	*blokfluit*	*polyester*	*prei*	*uitlaatgas*
(butterfly)	(recorder)	(polyester)	(leek)	(exhaust fumes)

*schaap*	*tamboerijn*	*satijn*	*pruim*	*parfum*
(sheep)	(tambourine)	(satin)	(plum)	(perfume)

*spin*	*fluitje*	*schuimrubber*	*selderij*	*shampoo*
(spider)	(whistle)	(foam rubber)	(celery)	(shampoo)

*stier*	*trombone*	*sokken*	*sinaasappel*	*sigaar*
(bull)	(trombone)	(socks)	(orange)	(cigar)

*varken*	*trompet*	*zeemlap*	*spinazie*	*tabak*
(pig)	(trumpet)	(chamois)	(spinach)	(tobacco)

*wesp*	*viool*	*vacht*	*tomaat*	*wierook*
(wasp)	(violin)	(fur)	(tomato)	(incense)

*zeester*	*xylofoon*	*zijde*	*wortel*	*zwavel*
(starfish)	(xylophone)	(silk)	(carrot)	(sulphur)

**Table 3.** Semantic categories with average and standard deviation (in brackets) of sensory modality strength ratings taken from Speed and Brysbaert (2022), word frequency (SUBTLEX), word length, and modality exclusivity (0 = completely multimodal, 1 = completely unimodal) across semantic categories.

	Animals (Vision)	Instruments (Audition)	Tactile objects (Haptic)	Food (Gustation)	Odor objects (Olfaction)
Vision	4.16	3.33	3.36	3.94	2.26
	(.49)	(.60)	(.36)	(.36)	(.93)

Audition	1.64	4.23	0.37	0.06	0.33
	(1.32)	(.34)	(.43)	(.13)	(.36)

Haptic	2.06	2.47	3.84	2.75	1.28
	(.71)	(.61)	(.40)	(.58)	(.87)

Interoception	0.27	0.28	0.33	0.24	0.28
	(.54)	(.33)	(.33)	(.35)	(.26)

Gustation	0.76	0.08	0.06	4.59	1.02
	(1.06)	(.14)	(.09)	(.33)	(1.25)

Olfaction	0.89	0.12	0.62	2.98	3.83
	(.94)	(.10)	(.62)	(.53)	(.51)

Word frequency	261.7	759.15	137.75	97.6	234.9
	(272.3)	(3032.3)	(251.9)	(80.12)	(341.3)

Word length	6.00	7.15	7.85	6.60	7.05
	(1.65)	(2.41)	(2.54)	(1.90)	(2.06)

Modality Exclusivity	0.46	0.41	.45	.32	.43
	(.15)	(.04)	(.05)	(.02)	(.10)

One-way ANOVAs with sensory modality as between items factor confirmed that items did not differ on word frequency (SUBTLEX Zipf values per billion words; Brysbaert et al., 2016) across semantic categories, *F*(4, 95) = .75, *p* = .56, *η*_p_^2^ = .031, and word length, *F*(4, 95) = 2.06, *p* = .09, *η*_p_^2^ = .073.

Sound files (object nouns and instructions) were recorded by a female native speaker of Dutch in a sound-attenuated booth using Adobe Audition CC 2018 at a sampling rate of 44000 Hz, in stereo and with a bit depth of 16 bits. The recordings were automatically processed in Python (version 3.6.6; Van Rossum & Drake, 2009) with pydub (version 0.24.1; Robert, et al., 2018) to splice out silences and equalize volume then checked in Audition to remove any remaining background noises using the Auto Heal function.

### Design

The study had a 6 by 2 mixed design, with the within-participants/within-item factor sensory modality (vision, audition, interoception, haptic, gustation, and olfaction) and the between-participants/within-items factor visual status (blind, sighted-controls).

### Procedure

The experiment was conducted online using Gorilla Experiment Builder (www.gorilla.sc) (Anwyl-Irvine et al., 2020). After giving informed consent participants completed a sound check and checked their browser allowed automatic playing of the audio stimuli. Participants were told to wear headphones. For blind participants, a quick screen reader compatibility check followed, as parts of the experiment required them to switch between different modes of their screen reader. Participants then completed the sensory ratings task and then answered some questions about the experiment and their experience with it. Sighted participants also completed a brief demographics questionnaire.

In the sensory rating task, participants were asked to indicate to what extent people experience each object by means of seeing, hearing, feeling, tasting, smelling and sensations within the body. This instruction departs from the original instructions of Lynott et al. (2020) where the second person pronoun was used (i.e., participants were asked to what extent “you” experience objects in relation to perceptual modalities). We did this because we wanted to assess blind participants’ conceptual representations rather than their own personal experience. We assumed the blind participants would rate all items as 0 on the visual scale if the question was about their own experience. Results from a pilot study in English indicated that the type of instruction did not affect the dominance of perceptual experience for each semantic category (see Supplementary Material S1) and, ratings in both instruction conditions were strongly correlated across perceptual modalities (all *r* > .97). Participants rated their sensory associations on a scale from 0 (not at all) to 5 (very strongly) followed by their familiarity with the object the word refers to on a scale from 1 (not at all familiar) to 5 (very familiar). They were instructed to press 0 if they misheard or did not know the word.

All stimuli and cues were presented auditorily in Dutch. Each trial started with a fixation beep presented for 227 ms, followed by 773 ms of silence, followed by the noun. Each noun was presented for the duration of the sound recording (between 600 ms and 1097 ms) and was followed by 1,000 ms silence. Each sensory modality cue was then presented consecutively, starting with vision (“*door zien*”), then audition (“*door horen*”), haptic (“*door voelen*”), gustation (“*door proeven*”), olfaction (“*door ruiken*”;), and finally interoception (“*door sensaties binnen het lichaam*”). Participants responded by pressing the number key associated with their rating. There was a period of 1,000 ms silence between producing the response and the next sensory modality being cued. After participants provided a response for interoception, there was again a 1,000 ms period of silence, followed by the cue to rate familiarity (“*bekendheid*” in Dutch). The intertrial interval was 2,500 ms.

The sensory ratings task was divided into two parts of each approximately 30–35 minutes completed on separate days. Each part started with the same instructions and a practice block of 4 trials (different items in each part). The purpose of the practice block was to ensure that participants understood the instructions. Should they have encountered problems they had the possibility to call the researcher. The 100 experimental trials were randomly assigned to each part; the 50 trials per part were presented in a different random order for each participant, with a self-paced break after every 10 trials. The instructions for the early-blind and sighted participants were identical, except that blind participants received a reminder to turn their screen reader off during the trials (due to potential conflicts arising from the response keys also being command keys for the screen reader) and that sighted participants were warned there will be nothing shown on screen during the trials (which might otherwise make them think the experiment had crashed). A summarized version of the instructions was presented after the practice block and each break to remind participants what they were supposed to do. Once a participant had completed the first part of the task, they had to wait until the next morning at 8am before they could begin the second part. Participants were encouraged not to wait longer than 72 hours before completing the second part.

### Data Analysis

Word ratings were excluded on a participant basis if participants rated the item’s familiarity as 0. We conducted linear mixed effects models with participants and items modelled as random effects in R (R Core Team, 2020) using the packages *lme4* (Bates et al., 2015) and *lmerTest* to retrieve *p*-values (Kuznetsova et al., 2017). The models would only converge with participants and items as random intercepts. We tested the effects of visual status (blind vs. sighted), sensory modality (vision, audition, interoception, haptic, gustation, and olfaction) and their interaction as fixed factors on the raw sensory ratings. Statistical significance was assessed by comparing models with and without the factor of interest using likelihood ratio tests with Chi square. We used effect coding for the factor visual status (0.5, −0.5) so that parameter estimates reflect the difference between the two levels. We followed up any significant interactions by testing the effect of visual status separately for each sensory modality using the *emmeans* package (Lenth, 2022), applying a Bonferroni correction for the number of follow-up models (i.e., *p* = .008 which is .05 divided by six sensory modalities).

## RESULTS

Intraclass correlation (ICC2k) ranged from good to excellent^1^: blind participants vision = .87, audition = .99, haptic = .94, interoception = .79, gustation = .99, olfaction = .99; sighted participants vision = .93, audition = .97, haptic = .92, interoception = .74, gustation = .98, olfaction = point = .98.

**Figure 1.**
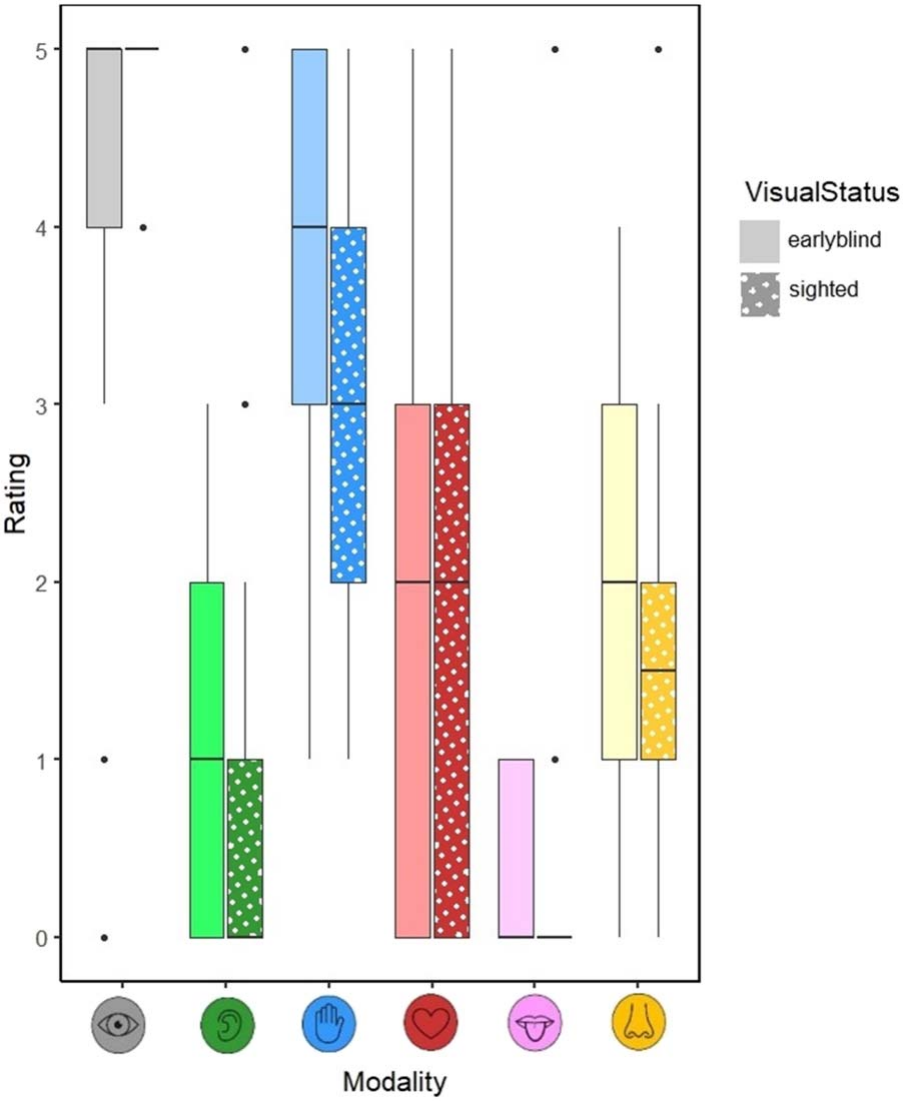
Median sensory ratings across semantic categories for vision, audition, haptic, interoception, gustation and olfaction (0 = not at all experienced in that sensory modality, 5 = very strongly experienced in that sensory modality). Whiskers indicate minimum and maximum values.

We found no main effect of visual status, *χ^2^*(1) = 1.28, *p* = .26, but a significant effect of sensory modality, *χ^2^*(1) = 5041, *p* < .001, demonstrating a clear visual dominance effect (see Figure 1). In addition, we found a significant interaction between visual status and sensory modality, *χ^2^*(5) = 112.69, *p* < .001. Follow-up contrasts revealed a significant effect of visual status for the haptic modality only, *β* = .30, *SE* = .08, *z* = 3.96, *p* < .001, with higher ratings for blind participants (*M* = 3.35, *SD* = 1.66) than sighted participants (*M* = 2.74, *SD* = 1.79). There was no difference between blind and sighted participants in ratings of the visual modality, *β* = .09, *SE* = .08, *z* = 1.15, *p* = .25 (blind participants: *M* = 4.09, *SD* = 1.55; sighted participants: *M* = 4.36, *SD* = 1.29). Data is available here: https://osf.io/85jna.

Overall, the results showed no difference between blind and sighted participants in their visual associations, suggesting blind people’s conceptual representations are learned indirectly, for example via language. On the other hand, we did find higher ratings of haptic associations in blind participants than sighted participants, implying sensory compensation.

**Figure 2.**
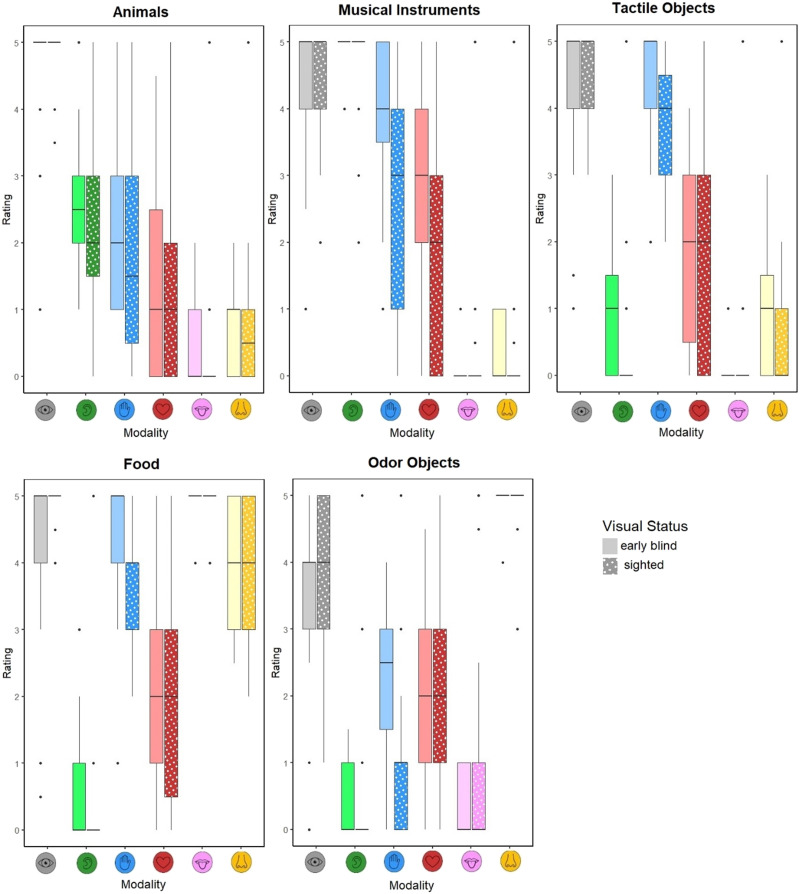
Median sensory ratings for vision, audition, haptic, interoception, gustation and olfaction (0 = not at all experience in that sensory modality, 5 = very strongly experience in that sensory modality) across semantic categories, animals, musical instruments, tactile objects, food, and odor objects. Whiskers indicate minimum and maximum values.

**Figure 3.**
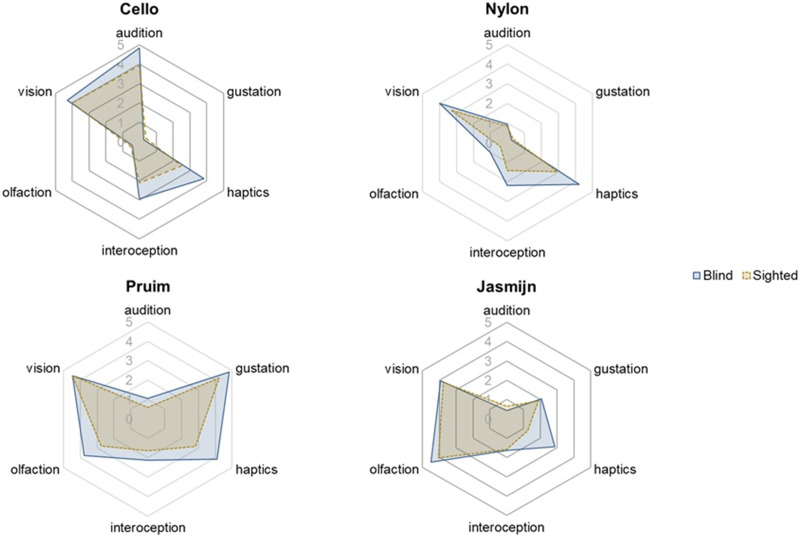
Spider plots of mean ratings in each modality in blind participants (blue) and sighted participants (yellow) for the words cello, nylon, pruim (prune) and jasmijn (jasmine).

It has been suggested that patterns of sensory association differ across semantic categories (Banks & Connell, 2023) Therefore, analyzing the data only by collapsing across semantic categories may hide differences between groups for other sensory modalities. We therefore conducted exploratory analyses assessing the interaction between visual status and sensory modality separately for each semantic category: animals, musical instruments, tactile objects, food, and odor objects. Analyses by semantic category broadly showed the same pattern of responses as in the main analysis (see Supplementary Analyses S2). Across semantic categories there was no difference between blind and sighted ratings for the visual modality. For all semantic categories except animals, haptic ratings were higher for blind than sighted participants (see Figure 3 for example words from each semantic category). In addition, musical instruments were rated as having stronger associations with interoception in the blind than sighted group (see Figure 2).

To further explore the similarity in ratings between groups, we calculated the correlation between mean item ratings of blind and sighted participants across semantic categories using Spearman’s correlation (due to non-normal distribution of the data). Ratings correlated significantly for all sensory modalities, but the correlation was lowest for vision (see Table 4). So although the visual ratings of the two groups were similar overall, there were a few words where the difference in mean rating was greater than 1. For example, blind participants rated the words *perfume* (*parfum*, blind *M* = 2.12, sighted *M* = 3.47) and *deodorant* (*deodorant*, blind *M* = 2.88, sighted *M* = 4) lower on vision than sighted participants. This may be because blind individuals have limited experience with the visual design of perfume and deodorant packaging. On the other hand, blind participants rated *satin* (*satijn*, blind *M* = 4.25, sighted *M* = 3.12) and *kerosine* (kerosine, blind *M* = 2.47, sighted *M* = 0.84) as more visual than sighted participants.

**Table 4.** Spearman correlation between blind and sighted ratings for each sensory modality.

	Spearman correlation (*r*)
Vision	.55
Audition	.83
Haptics	.93
Interoception	.68
Gustation	.75
Olfaction	.94

## GENERAL DISCUSSION

We investigated the role of direct perceptual experience in conceptual representation by collecting ratings of sensory associations of word meanings in blind and sighted individuals. In all but one semantic category, blind participants reported higher haptic ratings than sighted participants, even when haptic information was not particularly salient (e.g., for odor objects). This suggests an effect of crossmodal compensation in conceptual representation, where associated modalities may help to scaffold concepts when perceptual information from one modality is missing (see Speed & Majid, 2020). This enhanced haptic association may reflect the everyday dominance of haptic experience for blind individuals. Previous studies have found enhanced spatial acuity in blind people, driven by a reliance on tactile information (Wong et al., 2011). In word meaning studies, a significant positive correlation between visual and haptic ratings have been found (Lynott et al., 2020; Speed & Brysbaert, 2022; Speed & Majid, 2017). For example, in Speed and Brysbaert’s (2022) ratings of over 24,000 Dutch words, the correlation between visual and haptic ratings was *r* = .56. Because of this strong correlation, haptic information could be a good source of information to rely on when visual information is absent. That we did not find haptic compensation in the animal category, which is the semantic category where vision is most relevant, could be explained by the fact that only 2 out of 20 animals in our stimuli were animals typically kept as pets. Participants are unlikely to have had much haptic experience with the other animals (e.g., alligator, beaver, stork).

The correlation between sensory modalities may also explain why we did not observe higher ratings in other sensory modalities putatively enhanced in blind people i.e., audition and olfaction: they provide little compensatory information in the absence of vision. Notably, Amenta et al. (2024) observed higher ratings in early deaf individuals compared to hearing individuals across all sensory modalities except audition, but it is unlikely that all perceptual modalities compensate for lack of auditory experience (for example, odor will rarely be informative about sound). Instead, deaf individuals’ ratings may be higher because they compare their intact sensory experience with their lack of auditory experience. In other words, their reduced auditory experience may make other sensory experience more salient.

Surprisingly, we found no evidence of a difference in ratings of visual association between blind and sighted participants. Thus, having no experience of visual perception (apart from a few participants able to distinguish light and dark), does not appear to affect the strength of visual information underlying word meaning. This is in line with previous studies showing that blind and sighted individuals have similar semantic knowledge about visual properties (Bedny et al., 2019; Bedny & Saxe, 2012; Kim et al., 2019a; Landau & Gleitman, 1988; Petilli & Marelli, 2024). Moreover, mean visual ratings were always comparable between the two groups, and sometimes even higher for blind participants (e.g., for food concepts). This raises a potential concern that there may have been a ceiling effect; however, we also fail to observe a difference in ratings for concepts in the semantic category of odor objects, where average visual ratings are not very high (3.19 on a 0–5 scale), and therefore not at ceiling. The correlation in ratings between blind and sighted participants suggests there may indeed be a subtle effect of the lack of visual experience on conceptual representation, since the correlation between groups was lowest for the visual modality. This lower correlation appears to be caused by a few specific items, suggesting some conceptual information is learned via non-perceptual routes and may lead to fine-grained differences.

Taken together, the findings support the proposal that conceptual information about vision does not require direct perceptual experience to be learned, but can be acquired indirectly from other sources, such as language. The role of linguistic information for visual concepts in blind people has been shown with brain imaging, where color and action concepts activate temporal regions of the brain in blind participants, and posterior occipital cortex (an area involved in low-level visual perception) in sighted participants (Bottini et al., 2020). Some studies suggest patterns of responses in blind participants can be predicted by co-occurrence statistics in language input (Anceresi et al., 2023; Lewis et al., 2019; Ostarek et al., 2019; see Kim et al., 2019b, 2019c for an alternative view). In this vein, Günther et al. (2022) provide a model where visual images for objects never seen before are predicted based on existing relations between distributional linguistic information (from text corpora) and visual information (from an image database). Critically, however, the model possessed some pre-existing visual knowledge. The specific mechanism through which visual information is learned from linguistic input without any existing visual experience is still unclear and calls for further investigation.

In contrast to our study, a recent study with early deaf and hearing individuals did find a difference in the rated auditory strength of words, with deaf individuals rating words lower for their auditory association than hearing individuals (Amenta et al., 2024). This might suggest that it is more difficult to learn about auditory than visual properties from non-perceptual input, such as language, or other sensory information, like haptics. However, a critical methodological difference exists between Amenta et al. (2024) and the present study: we asked participants to indicate to what extent **people** experience each object through the senses; however Amenta et al. (2024) asked for a first person perspective by asking: “To what extent can **you** experience [WORD] by…” (emphasis in bold added).This may have lead to participants focus on their own experience, i.e., autobiographical associations, rather than relying on semantic associations more generally.

Since haptic compensation cannot occur for all concepts (e.g., animals that cannot physically be interacted with), the similarities between blind and sighted individuals makes salient the role of language as a possible source of conceptual information. Linguistic distributional information has been shown to predict the sensory modality of words, but at a coarse level. For example, Louwerse and Connell (2011) used linguistic context (word co-occurrences) in a large corpus of English to predict the sensory modality of words (as rated by humans in Lynott & Connell, 2009). Instead of the five sensory modalities included in the original ratings, a three-factor structure was found to underlie the co-occurrence data. Particularly relevant for the present study is the first component extracted which correlated significantly with visual and haptic ratings. This means that information extracted from language may imply both visual and haptic modalities, which could also explain the higher haptic ratings by the blind group in our data.

The results of this study are based on explicit ratings and therefore cannot speak to whether sensory information underlying word meaning is recruited online during language processing for blind people. In sighted individuals, sensory ratings—such as those collected here—predict lexical decision time and word naming (Connell & Lynott, 2012, 2016; Speed & Brysbaert, 2022; Speed & Majid, 2017), but it is unknown whether this would also be the case for blind individuals. The idea is plausible. When a sensory modality is lost, it can affect online lexical processing. For example, a patient with damage to the auditory association cortex has been shown to be impaired when processing sound-related words compared to non-sound-related words (Trumpp et al., 2013). On the other hand, Bottini et al. (2022) found blind participants showed a concreteness advantage in a lexical decision task, an effect typically thought to be driven by visual simulation. Later analyses demonstrated that linguistic distributional statistics predicted responses in the task better for blind than sighted participants (Anceresi et al., 2023). This suggests relying on linguistic associations instead of visual simulation is not detrimental to lexical processing. Further work could disentangle the separate roles played by linguistic information and the compensatory haptic information blind people appear to rely on.

In sum, our results provide new evidence that blind participants learn about the sensory underpinnings of words through sensory experience, to some extent. While lack of visual experience does not affect the visual content of conceptual representations, it does lead to compensation in another correlated sensory modality, mirroring perceptual experience in blind individuals. These findings support hybrid models of semantics, where perceptual and linguistic information are critical in word meaning (Connell, 2019; Louwerse, 2011).

## Acknowledgments

We would like to thank Dermot Lynott and the anonymous reviewers for comments on the manuscript.

## Funding Information

This work was supported by Radboud University research funds to L.S. and a start-up grant from the University of York to A.M.

## Author Contributions

L.S.: Conceptualization; Formal analysis; Funding acquisition; Methodology; Project administration; Visualization; Writing – original draft. E.D.P.: Investigation; Methodology; Project administration. T.P.D.: Investigation. H.B.: Conceptualization; Methodology. A.M.: Conceptualization; Funding acquisition; Methodology; Supervision; Writing – review & editing.

## Data Availability Statement

Data and analysis scripts are available here: https://osf.io/85jna.

## Note

^1^ Due to some participants’ missing data on single items, ICC was calculated used 85 items for the blind group and 89 items for the sighted group.

## Supplementary Material


