# Supplementary material for: Haptic Compensation in Blind People’s Conceptual Representations
Source: Open Mind (Camb). 2025 Oct 17;9:1786–801. doi: 10.1162/OPMI.a.250 (PMC12618011; doi:10.1162/OPMI.a.250)
Supplement: Supplementary file 1 [file opmi-09-1786-s001.docx]

 Haptic compensation in blind people’s conceptual representations

**Supplementary Analyses**

**S1. Pilot data**

**Method**

*Participants*

One-hundred participants took part in the study, recruited through Prolific Academic. One participant had problems with the online survey and failed to complete the questionnaire. This left 99 participants (55 female) with mean age 37.22 (*SE* = 1.30). All participants were native speakers of English and were born and grew up in the United Kingdom. They were paid £4.50 for their participation.

*Materials*

20 English words per modality (vision, audition, haptic, gustation, olfaction) were selected from the Lancaster Sensorimotor Norms (Lynott, Connell, Brysbaert, Brand, & Carney, 2019) based on their dominant modality. Items were selected so that they fit into the following superordinate categories corresponding to each modality: animals (vision), musical instruments (audition), fabrics (haptics), food (gustation), and odorous objects (olfaction).

One-way ANOVAs with modality as a between items factor confirmed that items did not differ on word frequency (SUBTLEX), *F*(4, 95) = 1.69, *p* = .16, η^2^_p_ = .066, and word length, *F*(4, 95) = .79, *p* = .53, η^2^_p_ = .032.

Table 1. Words selected for each semantic category.

| Animals  (Vision) | Instruments (Audition) | Tactile objects  (Haptics) | Food  (Gustation) | Odor objects  (Olfaction) |
| --- | --- | --- | --- | --- |
| caterpillar  donkey  sheep  beetle  frog  rabbit  pheasant  badger  alpaca  lobster  deer  dove  goat  rooster  starfish  bull  fox  hedgehog  wasp  spider | banjo  cello  clarinet  drum  guitar  recorder  harp  piano  saxophone  tambourine  violin  flute  trumpet  bell  accordion  bagpipes  chime  viola  fiddle  ukulele | coat  glove  sweater  rucksack  blanket  cushion  scarf  silk  sponge  velvet  wool  fleece  quilt  cotton  mattress  handkerchief  carpet  spandex  pillow  sheepskin | banana  broccoli  cabbage  grape  kiwi  lemon  onion  peach  pineapple  papaya  raspberry  spinach  tomato  asparagus  avocado  carrot  mango  orange  plum  celery | cigarette  cigar  jasmine  smoke  lavender  diesel  bleach  musk  gas  manure  perfume  deodorant  methane  petrol  sulphur  incense  cologne  aftershave  hairspray  disinfectant |

Words were recorded by a native speaker of British English using Audacity. Sound files were centered, bandpass filtered between 50HZ and 10kHz with a smoothing width of 100Hz, and RMS equalized to 0.09 Pa.

*Procedure*

The questionnaire was created with Qualtrics. Participants were presented with each word separately in a random order, and had to click on a “play” icon to hear each word. Participants were randomly allocated to one of two instruction groups. They were either asked “To what extent do you experience the meaning of this word…” (You group; *N* = 51) or “To what extent do people experience the meaning of this word…” (People group; *N* = 48), followed by a 0 (not at all) to 5 (greatly) rating scale for five perceptual modalities: feeling through touch, hearing, seeing, tasting, smelling. They were then asked to rate how familiar they were with the meaning of each word on a scale of 0 (very unfamiliar) to 5 (very familiar). Participants made their response by clicking on a number on the scale. The list of words was split into two sets to allow for a break halfway through the questionnaire. The order of presentation of each set was counterbalanced across participants. Participants completed a practice trial before beginning the full questionnaire.

At the end of the questionnaire participants were asked for demographic information, and asked what strategy they used to complete the ratings: either “I rated the words in the order of perceptual senses they were presented in i.e., beginning with “by feeling through touch””, “I rated the words by beginning with the perceptual senses that a word was most strongly experienced in”, or “Other” (allowing an additional free response).

**Results**

Sixty-five participants (66%) reported that they “rated the words in the order of perceptual senses they were presented in i.e., beginning with “by feeling through touch”, 30 participants (30%) reported that they “rated the words by beginning with the perceptual senses that a word was most strongly experienced in”, and 4 responded “Other”. Two participants reported that they used a mixture of both strategies.

All items were rated as familiar (familiarity > 3.90). No participant had low mean familiarity ratings or unusual patterns of response. For further analyses, ratings of perceptual strength were averaged over participants for each word and perceptual modality

*Were individual items rated as dominant in same modality?*

Words were categorized into their dominant modality according to the modality that received the highest mean rating. Combining both instruction groups, there were 8 words categorized differently to their categorization in the Lancaster Sensorimotor Norms (Lynott, Connell, Brysbaert, Brand, & Carney, 2019):

rooster (auditory instead of vision)

carpet (vision instead of touch)

coat (vision instead of touch) [In “You” this was dominant in touch]

handkerchief (vision instead of touch)

rucksack (vision instead of touch)

scarf (vision instead of touch)

spandex (vision instead of touch)

sweater (vision instead of touch)

Additionally in the “people” condition only:

glove (vision instead of touch)

*Do categories differ in perceptual strength?*

To check that words in the semantic categories were rated stronger in their assigned modality (i.e., animal category rated higher on vision), one-way ANOVAs were conducted for each modality rating scale separately, testing for the effect of semantic category. We first conducted these analyses for the whole dataset, followed by separately per type of instruction.

*Full dataset*

Analyses confirmed that our semantic categories were indeed rated most strongly in the intended sensory modality. There was a significant effect of category on visual ratings, *F*(4,95) = 28.77, *p* < .001. As expected, ratings of visual strength were higher for animals than musical instruments, *p* = .003, odor objects, *p* < .001, and tactile objects, *p* = .02, but only marginally higher than food, *p* = .07. There was also a significant effect of category on auditory ratings, *F*(4,95) = 166.80, *p* < .001, with ratings of auditory strength higher for musical instruments than all other semantic categories, *p* < .001, a significant effect of category on olfactory ratings, *F*(4,95) = 379.91, *p* < .001, with ratings of olfactory strength higher for odor objects than all other semantic categories, *p* < .001, a significant effect of category on gustatory ratings, *F*(4,95) = 379.91, *p* < .001, with ratings of gustatory strength higher for food than all other categories, *p* < .001, and a significant effect of category on haptic ratings, *F*(4,95) = 95.17, *p* < .001, with ratings of haptic strength higher for tactile objects than all other categories, *p* < .001.

*“You” instruction*

Results for the “you” instruction confirmed the intended pattern, with semantic categories rated most strongly in the intended sensory modality. There was a significant effect of category on visual ratings, *F*(4,95) = 29.98, *p* < .001, with ratings of visual strength higher for animals than all categories, *p* < .001, a significant effect of category on auditory ratings, *F*(4,95) = 146.85, *p* < .001, with ratings of auditory strength higher for musical instruments than all other categories, *p* < .001, a significant effect of category on olfactory rating *F*(4,95) = 327.06, *p* < .001, with ratings of olfactory strength higher for odor objects than all other categories, *p* < .001, a significant effect of category on gustatory ratings, *F*(4,95) = 276.24, *p* < .00, with ratings of gustatory strength higher for food than all other categories, *p* < .001, and a significant effect of category on haptic ratings, *F*(4,95) = 94.77, *p* < .001, with ratings of haptic strength higher for tactile objects that all other categories, *p* < .001.

*“People” instruction*

Results for the “people” instruction generally followed the intended pattern, but less so for visual ratings. There was a significant effect of category on visual ratings, *F*(4,95) = 28.60, *p* < .001. Ratings of visual strength were higher for animals than musical instruments, *p* = .006, and odor objects, *p* < .001, but the comparison did not reach significance for tactile objects, *p* = .06, or food, *p* = .10. There was also a significant effect of category on auditory ratings, *F*(4,95) = 174.7, *p* < .001, with ratings of auditory strength higher for musical instruments than all other categories, *p* < .001, a significant effect of category on olfactory ratings, *F*(4,95) = 372.58, *p* < .001, with ratings of olfactory strength higher for odor objects than all other categories, *p* < .001, an effect of category on gustatory ratings, *F*(4,95) = 165.33, *p* < .001, with ratings of gustatory strength higher for food than all other categories, *p* < .001, and a significant effect of category on haptic ratings, *F*(4,95) = 86.92, *p* < .001, with ratings of haptic strength higher for tactile objects than all other categories, *p* <.001.

*Do ratings differ according to instruction?*

Ratings in both instruction conditions were strongly correlated across sensory modalities (see Table 2).

Table 2. Correlation between ratings in “You” and “People” condition

|  | Vision | Audition | Olfaction | Gustation | Haptic |
| --- | --- | --- | --- | --- | --- |
| Correlation | .97 | .99 | .99 | .99 | .97 |

In order to check whether the pattern of ratings differed depending on instruction, we conducted a mixed ANOVA on the perceptual ratings with instruction (people vs. you) and rating modality (vision, audition, olfaction, gustation, haptic) as the within items conditions and semantic category (animals, instruments, odor objects, food, tactile objects) as the between items condition.

There was a significant interaction between instruction and rating modality *F*(4, 380) = 136.30, *p* < .001, *n*_p_^2^ = .589. To follow-up the interaction we tested for the effect of instruction separately for each modality. There was a significant effect of instruction for ratings in all modalities. For all modalities except audition, ratings were higher with the “people” instruction than the “you” instruction. In contrast, for ratings of auditory strength, ratings were higher for the “you” instruction than the “people” instruction. In general the overall pattern of strength across sensory modalities was comparable across the two instruction conditions.


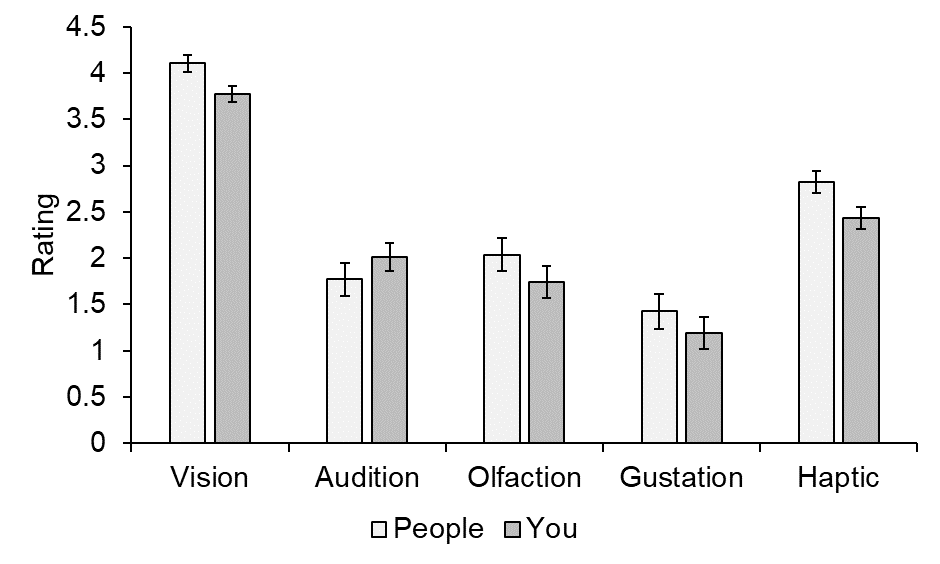


There was also significant interaction between instruction and semantic category, *F*(4, 380) = 8.41, *p* <.001, *n*_p_^2^ = .261. To follow-up the interaction we tested for the effect of instruction separately for each semantic category. In all semantic categories there was a significant effect of instruction, with ratings significantly higher with the “people” instruction than the “you” instruction. The general pattern of modality strength across semantic categories was comparable across instructions.


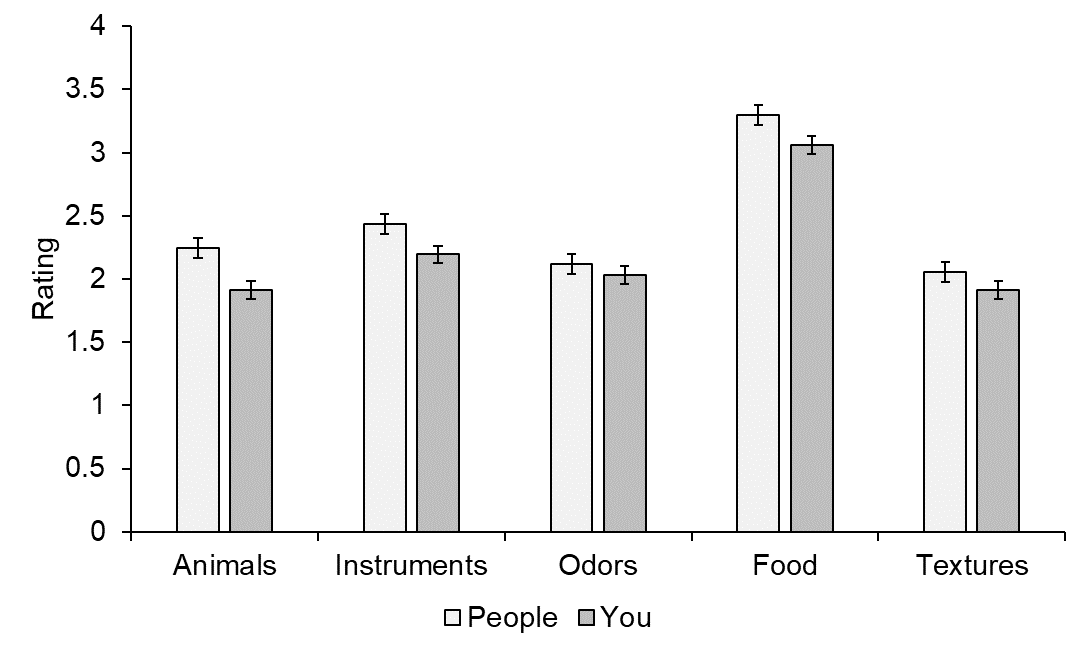


There was also a three-way interaction between instruction, ratings modality and semantic category, *F*(4, 380) = 18.15, p <.001, *n*_p_^2^ = .433. We therefore checked whether there was a difference between ratings in the “people” and “you” instruction condition within each modality, for each semantic category separately.

For animals, ratings were higher in the “people” condition than the “you” condition for all modalities except for audition. For instruments, there were higher ratings for “people” than “you” in all modalities. For food and odor objects, there were higher ratings for “people” than “you” in all modalities (vision *p* = .052 for odor objects) except audition, where instead ratings were higher with the “you” instruction than the “people” instruction. This pattern was the same for tactile objects where additionally there was no effect of instruction in gustatory ratings. Overall, the pattern of ratings across modalities for each semantic category was comparable in the “people” and “you” condition. Only ratings of auditory strength were affected differently.

**S2. Comparing blind versus sighted ratings separately by semantic category**

We assessed the effect of visual status, sensory modality, and the interaction between visual status and sensory modality separately for each semantic category, using the same data analysis procedures as described in the main paper.

*Animals*

For the animals category, there was no main effect of visual status, *χ^2^*(1) = .47, *p* = .50, but a significant effect of sensory modality, *χ^2^*(1) = 2358, *p* < .001, with ratings of vision the highest. There was also a significant interaction between visual status and sensory modality, *χ^2^*(5) = 28.52, *p* < .001. Follow-up contrasts revealed a significant effect of visual status for the auditory modality, β = .21, *SE* = .10, *z* = 2.05, *p* = .04, with blind individuals giving higher ratings than sighted individuals, but this did not meet our Bonferroni-corrected criteria of *p* = .008. Even though the animal category was the most visually-dominant of our semantic categories, there was no evidence that blind participant’s lack of visual experience meant they viewed animals as less “visual” than sighted participants, β = .15, *SE* = .10, *z* = 1.42, *p* = .16 (blind participants: *M* = 4.39, *SD* = 1.38; sighted participants: *M* = 4.69, *SD* = .79). Mean ratings are displayed in Figure 2 in the main manuscript.

*Musical instruments*

For musical instruments, there was again no main effect of visual status, *χ^2^*(1) = 2.91, *p* = .09, and again a main effect of sensory modality, *χ^2^*(1) = 4954, *p* < .001. Musical instruments were rated highly in the auditory and visual modalities (see Figure 2 main manuscript). There was also an interaction between visual status and sensory modality, *χ^2^*(5) = 104, *p* < .001, with follow-up contrasts revealing higher haptic ratings in the blind (*M* = 3.72, *SD* = 1.36) compared to sighted group (*M* = 2.88, *SD* = 1.74), β = .40, *SE* = .09, *z* = 4.65, *p* < .001, and higher ratings of interoception in the blind (*M* = 2.6, *SD* = 1.49) compared to sighted group (*M* = 1.99, *SD* = 1.65), β = .29, *SE* = .09, *z* = 3.38, *p* < .001. Again, there was no difference between the two groups in ratings of vision, β = .04, *SE* = .09, *z* = .44, *p* = .66 (blind participants: *M* = 4.29, *SD* = 1.34; sighted participants: *M* = 4.34, *SD* = 1.12).

*Tactile objects*

For tactile objects, there was no main effect of visual status, *χ^2^*(1) = 1.67, *p* = .20. There was a main effect of sensory modality, *χ^2^*(1) = 3709, *p* < .001, with highest ratings in the haptic and visual modality (see Figure 2 main manuscript). We again found an interaction between visual status and sensory modality, *χ^2^*(5) = 28.56, *p* < .001. Follow-up contrasts revealed higher haptic ratings in the blind group (*M* = 4.29, *SD* = 1.10) compared to the sighted group (*M* = 3.69, *SD* = 1.37), β = .30, *SE* = .10, *z* = 43.18, *p* = .002. There was no difference in visual ratings between the blind and sighted groups, β = .01, *SE* = .10, *z* = .10, *p* = .92 (blind participants: *M* = 4.3, *SD* = 1.36; sighted participants: *M* = 4.26, *SD* = 1.15).

*Food*

There was no main effect of visual status for ratings of food, *χ^2^*(1) = .24, *p* = .20, but there was a main effect of sensory modality, *χ^2^*(1) = 3910, *p* < .001, with high ratings in the gustatory and visual modalities (see Figure 2 main manuscript). There was again a significant interaction between visual status and sensory modality, *χ^2^*(5) = 68.61, *p* < .001. Follow-up contrasts revealed higher haptic ratings in the blind (*M* = 4.09, *SD* = 1.17) compared to sighted group (*M* = 3.52, *SD* = 1.24), β = .30, *SE* = .10, *z* = 3.18, *p* = .002. There also appeared to be a difference between the two groups in visual ratings, but this was not significant using the Bonferroni-corrected p-value of .008, β = .20, *SE* = .09, *z* = 2.09, *p* = .04 (blind participants: *M* = 4.36, *SD* = 1.31; sighted participants: *M* = 4.72, *SD* = .65).

*Odor objects*

For ratings of odor objects, there was no main effect of visual status, *χ^2^*(1) = .74, *p* = .39, but there was a main effect of sensory modality, *χ^2^*(1) = 2408, *p* < .001. The highest ratings occurred for the olfactory modality (see Figure 2 main manuscript). There was also a significant interaction between visual status and sensory modality, *χ^2^*(5) = 68.61, *p* < .001. Follow-up contrasts indicated a significant effect of visual status in the haptic modality, β = .39, *SE* = .09, *z* = 4.08, *p* < .001, with higher haptic ratings in the blind (*M* = 2.2, *SD* = 1.65) compared to sighted group (*M* = 1.44, *SD* = 1.65). There was again no effect of visual status on ratings of visual associations, β = .09, *SE* = .09, *z* = .90, *p* = .39 (blind participants: *M* = 3.1, *SD* = 1.87; sighted participants: *M* = 3.29, *SD* = 1.84).
